# Inpatient Characteristics and Outcomes of Venous Thromboembolism Among Children and Adolescents

**DOI:** 10.1001/jamanetworkopen.2026.17459

**Published:** 2026-06-09

**Authors:** Sabrina Friebe, Dierk Scheinert, Esra Tokur Sonuvar, Toralf Kirsten, Eva Freisinger

**Affiliations:** 1 Institute for Medical Informatics, Statistics and Epidemiology, Department of Medical Data Science, Leipzig University, Leipzig, Germany; 2 Department of Angiology, University Hospital Leipzig, Leipzig, Germany; 3 Faculty of Medicine, Westfaelische Wilhelms University Muenster, Muenster, Germany

## Abstract

**Importance:**

Endogenous venous thromboembolism (VTE), including deep vein thrombosis and pulmonary embolism (PE), is considered a rare event in pediatric patients, and its health care implications are largely unstudied. However, its burden on the health care system may be disproportionately higher owing to age-specific risk factors.

**Objective:**

To evaluate and characterize the inpatient cohort of children and adolescents with VTE and PE.

**Design, Setting, and Participants:**

This nationwide cohort study used data on inpatient cases of VTE in Germany (data source: Federal Bureau of Statistics DESTATIS) from 2020 to 2024. Cases younger than 20 years with VTE as the main or secondary diagnosis, as well as a subcohort with PE as the main diagnosis, were analyzed regarding risk profiles, inpatient care, and outcomes stratified by sex and age.

**Main Outcomes and Measures:**

Hospital-based incidence of VTE (VTE cases per 10 000 inpatient cases per year) and in-hospital mortality.

**Results:**

This study identified 14 108 pediatric inpatient cases of VTE (mean [SD] age, 9.0 [7.3] years; 7201 male [51.0%]), corresponding to a hospital-based incidence of 15.3 per 10 000 pediatric cases per year. A total of 3311 patients with VTE (23.5%), comprising 1361 of 6907 female patients (19.7%) and 1950 of 7201 male patients (27.1%), were infants younger than 1 year. Risk factors for VTE varied, as rates of infection, chronic organ failure, and congenital diseases decreased with increasing patient age, while cancer was most commonly diagnosed among cases aged 5 to 14 years, and thrombophilia remained a relatively constant risk factor across all age groups. PE occurred in 1564 VTE cases (11.1%); among cases whose main diagnosis was PE, 624 of 888 (70.3%) were female. The in-hospital mortality rate in the VTE cohort was 3.7% (522 of 14 108); mortality risk was significantly increased among infants aged 4 years or younger (OR, 3.52 [95% CI, 2.73-4.57]; *P* < .001).

**Conclusions and Relevance:**

This cohort study found a marked inpatient health care and economic burden for VTE among children and adolescents. Mortality risk was significantly increased among infants younger than 1 year. Studies are needed to provide evidence-based support for the safety and effectiveness of medical interventions for children and adolescents with VTE.

## Key Points

**Question:**

To what extent are venous thromboembolism (VTE) and pulmonary embolism in hospitalized pediatric patients associated with specific risks, treatments, and outcomes?

**Findings:**

This nationwide inpatient cohort study found 14 108 cases of VTE among children and adolescents, for a hospital-based incidence of VTE of 15.3 per 10 000 pediatric cases per year. A total of 23.5% of VTE cases occurred in infants younger than 1 year; these were associated with the highest health economic burden and were independently associated with increased in-hospital mortality.

**Meaning:**

This study suggests that the hospital-based incidence of VTE among pediatric patients is 30- to 100-fold increased compared with reported population-based incidence rates, and very young children are at particular risk of fatal outcomes.

## Teaser

This cohort study evaluates the incidence of venous thromboembolism and pulmonary embolism among hospitalized children and adolescents and characterizes their association with in-hospital mortality.

## Introduction

Venous thromboembolism (VTE), comprising deep vein thrombosis (DVT) and pulmonary embolism (PE), is a severe and potentially life-threatening condition. Unlike in adults, in whom VTE affects approximately 10 in 10 000 persons per year,^1,2^ VTE is considered a rare disease among children and adolescents; incidences of 1.4 to 4.9 in 100 000 children per year are reported in pediatric cohorts from epidemiologic studies.^3,4,5^ A relatively high occurrence of VTE is commonly observed among neonates (5.1 per 100 000 births^6^) compared with the low rates in early to middle childhood, followed by a second peak of disease during late adolescence.^7,8^ The reasons for these alternating trends in VTE morbidity remain largely unclear; however, the difference in age-related procoagulant factors is the subject of scientific discussion.

The pathophysiological basis for the development of VTE is a disorder of blood coagulation via the components endothelial damage, stasis of flow, and hypercoagulable state (Virchow triad^9^). Genetic disorders leading to impaired function or lack of function of anticoagulant active factors therefore increase the risk of VTE. Prominent examples of such inherited thrombophilia include protein S deficiency, protein C deficiency, antithrombin deficiency, factor V Leiden polymorphism, and prothrombin polymorphism.^9,10^ Apart from endogenous thrombophilia, several acquired factors, such as malignant diseases, organ dysfunction (eg, cardiomyopathy and kidney failure), and infectious diseases, are associated with increased coagulation and clot formation.^11^ Modifiable or exogenous risk factors of VTE include obesity; nicotine abuse, particularly in combination with hormone therapy; immobility, severe trauma or major surgery, and the introduction of foreign material in the blood vessel (eg, use of central vein catheter).^12^

The subordinate aim of VTE management is to prevent the development of severe complications such as potentially life-threatening PE, postthrombotic syndrome, or recurrent thromboembolic events. The management of VTE in pediatric patients includes anticoagulation and local or systemic thrombolysis, as well as catheter-directed thrombectomy for high-risk cases.^13,14^ Given the lack of original research and barely existent registries for VTE in children and adolescents, the choice of therapeutic management is difficult. The recommendations of the few existing guidelines on VTE in pediatric patients are based mostly on extrapolated studies of adult populations, resulting in correspondingly weak grades of recommendation.^13^ In particular, the lack of valid prospective studies on the safety and effectiveness of drug therapies for children and adolescents results in reliance on off-label experience. Treatment options may also be limited due to a lack of expertise or the unavailability of invasive procedures (eg, extracorporeal membrane oxygenation for children).

In summary, there are no uniform standards of care for VTE and PE management for children and adolescents. Nevertheless, therapeutic decisions must be made with every medical consultation, whether caused by or presenting with VTE, to avert complications and sequelae.

As a first step, this study aims to record the actual in-hospital incidence of VTE at the federal level and, furthermore, to characterize VTE with regard to anatomic region, risk factors in terms of age and sex, inpatient treatment, the occurrence of PE, and in-hospital mortality.

## Methods

### Data Source

Data for this cohort study were obtained from the national inpatient database of the Research Data Centers of the Federal Statistical Office of Germany (DESTATIS). The dataset includes all inpatient cases recorded annually, excluding outpatient treatments and psychiatric or psychosomatic units. Each case contains 1 principal diagnosis representing the main reason for hospital admission, as well as multiple secondary diagnoses, reflecting case complexity. Diagnoses are coded according to the *International Statistical Classification of Diseases and Related Health Problems, 10th Revision, German Modification* (*ICD-10-GM*) classification. In addition, the dataset provides information on procedures, health care structures, length of stay, and costs. Procedures are coded using the German Operation and Procedure Classification (OPS); both *ICD-10-GM* and OPS are subject to annual updates. As reporting to DESTATIS is legally required for reimbursement purposes, the dataset can be considered comprehensive. The following data source of the DESTATIS has been used: Research Data Centers of the Federal Statistical Office and Statistical Offices of the Federal States.^15,16,17,18,19^ Comprehensive review by an ethics committee of the use of these fully anonymized DESTATIS data has been waived by the ethics committee pursuant to § 15 MusterBOÄrztInnen; § 15 BO SLÄK, Saxony, Germany. DESTATIS approved all analysis scripts regarding compliance with confidentiality and data protection regulations prior to publication. The results reporting complied with Strengthening the Reporting of Observational Studies in Epidemiology (STROBE) reporting guideline.

### Cohort Formation

VTE was defined as DVT in various anatomical locations (*ICD-10-GM* codes I80-82, I63.6, I67.6, and G08), including catheter-associated thrombosis (*ICD-10-GM* code T82.8) and PE (*ICD-10-GM* codes I26.0 and I26.9). Common risk factors for VTE (eg, cancer and thrombophilia [consisting of primary thombophilia, other thrombophilia, disseminated intravascular coagulation, and hyperhomocystheinemia], as well as obesity, heart failure, chronic kidney disease, sepsis, multiple trauma, congenital venous malformation, or chromosomal abnormalities) have been assessed for further analysis. Procedural codes were obtained for intensive care, invasive ventilation, systemic thrombolysis, and endoluminal procedures to treat PE. In-hospital mortality and complications, such as acute cor pulmonale, heart failure, resuscitation, or major bleeding, served as outcome parameters. Furthermore, economic data such as costs per case and length of hospital stay were obtained as far as available. The eTable in Supplement 1 presents all *ICD-10-GM* and OPS codes that have been used for selection and further stratification of the cohort.

For the study, all inpatient cases with a main or secondary diagnosis of VTE (DVT and/or PE) and with patient age younger than 20 years at the time of referral were requested for the data years 2020 through 2024. These requests resulted in 14 108 VTE cases; from this cohort, a subcohort consisting of 888 inpatient cases with PE (as the main diagnosis) was formed to assess just PE-specific treatment and outcomes. For further information on case selection and missing values, see the flowchart in eFigure 1 in Supplement 1.

### Statistical Analysis

Observational data on trends over time for VTE and PE were stratified by sex and age groups. Characterization of the cohorts comprised common predisposing risk factors for VTE, sex, and age categories. To test for statistical significance between the groups (eg, male vs female), the χ^2^ test was used for categorical variables, and the Mann-Whitney test was used for quantitative variables. The complete VTE cohort was included in logistic regression analyses. Two separate models were specified, with the dependent variables defined as (1) the occurrence of PE, coded as either a primary or secondary diagnosis, and (2) in-hospital mortality. Covariates included age group, sex, and relevant comorbidities. These models provide adjusted estimates of associations while acknowledging that causal inference cannot be formally established. All analyses were conducted using R statistical software (RStudio, version 2025.05.0 Posit Software, PBC; R Project for Statistical Computing). All *P* values were from 2-sided tests and results were deemed statistically significant at *P* < .05.

## Results

### VTE Cohort

In the nationwide inpatient sample, 14 108 VTE cases (mean [SD] age, 9.0 [7.3] years; 7201 male [51.0%] and 6907 female [49.0%]) in children and adolescents younger than 20 years were identified from 2020 to 2024 (Table 1),^15,16,17,18,19^ corresponding to a hospital-based incidence of VTE of 15.3 per 10 000 pediatric inpatient cases per year. Case numbers remained relatively stable over the data period (eFigure 2 in Supplement 1). The median age in the VTE cohort was 12 years (IQR, 3-18 years) for female cases vs 7 years (IQR, 0-15 years) for male cases (Table 1).^15,16,17,18,19^ A total of 3311 patients with VTE (23.5%), comprising 1361 of 6907 female patients (19.7%) and 1950 of 7201 male patients (27.1%), were infants younger than 1 year (*P* < .001). Whereas among infants and children, there was a clear predominance of male inpatients with VTE, among adolescents, most VTE and PE diagnoses occurred among female inpatient cases (eFigure 3A and B in Supplement 1).

**Table 1.** Baseline Characteristics of Patients With VTE and PE^a^

Characteristic	VTE				PE (main diagnosis)			
	Female	Male	*P* value	Total	Female	Male	*P* value	Total
VTE, No. (%)^b^	6907 (49.0)	7201 (51.0)	NA	14 108 (100)	624 (70.3)	264 (29.7)	NA	888 (100)
VTE characteristics								
DVT lower extremity, No. (%)	1546 (22.4)	1188 (16.5)	<.001	2734 (19.4)	NA	NA	NA	NA
DVT upper extremity, No. (%)	1037 (15.0)	1137 (15.8)	.21	2174 (15.4)	NA	NA	NA	NA
DVT inferior vena cava, No. (%)	649 (9.4)	680 (9.4)	.95	1329 (9.4)	NA	NA	NA	NA
DVT visceral veins, No. (%)	767 (11.1)	884 (12.3)	.03	1651 (11.7)	NA	NA	NA	NA
DVT other veins, No. (%)	339 (4.9)	416 (5.8)	.02	755 (5.4)	NA	NA	NA	NA
DVT intracranial, No. (%)	1085 (15.7)	1279 (17.8)	.001	2364 (16.8)	NA	NA	NA	NA
DVT catheter associated, No. (%)	1338 (19.4)	1702 (23.6)	<.001	3040 (21.5)	NA	NA	NA	NA
Postthrombotic syndrome, No. (%)	15 (0.2)	8 (0.1)	.18	23 (0.2)	NA	NA	NA	NA
DVT alone, No. (%)	6247 (90.4)	6791 (94.3)	<.001	13 038 (92.4)	NA	NA	NA	NA
PE, No. (%)^b^	948 (13.7)	616 (8.6)	<.001	1564 (11.1)	NA	NA	NA	NA
PE as main diagnosis, No. (%)	624 (9.0)	264 (3.7)	<.001	888 (6.3)	NA	NA	NA	NA
Patient characteristics								
Age, mean (SD), y	9.9 (7.3)	8.0 (7.1)	NA	9.0 (7.3)	17.3 (2.8)	16.2 (4.2)	NA	17.0 (3.3)
Age, median (IQR), y	12 (3-18)	7 (0-15)	<.001	9 (1-15)	18 (17-19)	18 (16-19)	.13	18 (16-19)
Cases aged <1 y, No. (%)	1361 (19.7)	1950 (27.1)	<.001	3311 (23.5)	8 (1.3)	9 (3.4)	.06	17 (1.9)
Codiagnoses								
Obesity, No. (%)	170 (2.5)	124 (1.7)	.003	294 (2.1)	54 (8.7)	33 (12.5)	.10	87 (9.8)
Cancer, No. (%)	891 (12.9)	115 (16.0)	<.001	2042 (14.5)	<3^c^	6^c^	NA	8 (0.9)
Thrombophilia, No. (%)	628 (9.1)	677 (9.4)	.55	1305 (9.3)	46 (7.4)	49 (18.6)	<.001	95 (10.7)
Kidney insufficiency, No. (%)	208 (3.0)	241 (3.3)	.28	449 (3.2)	6^c^	<3^c^	NA	7 (0.8)
Heart failure, No. (%)	498 (7.2)	559 (7.8)	.22	1057 (7.5)	53 (8.5)	21 (8.0%)	.89	74 (8.3)
Sepsis, No. (%)	328 (4.7)	421 (5.8)	.004	749 (5.3)	4^c^	<3^c^	NA	5 (0.6)
Polytrauma, No. (%)	4 (0.1)	9 (0.1)	.30	13 (0.1)	0	0	NA	0
Congenital venous malformation, No. (%)	199 (2.9)	200 (2.8)	.75	399 (2.8)	<3^c^	4^c^	NA	5 (0.6)
Chromosomal abnormalities, No. (%)	194 (2.8)	179 (2.5)	.25	373 (2.6)	7^c^	6^c^	NA	13 (1.5)
COVID-19, No. (%)	243 (3.5)	250 (3.5)	.92	493 (3.5)	44 (7.1)	24 (9.1)	.36	68 (7.7)
Economic data								
Length of in-hospital stay for VTE, median (IQR), d	7 (4-19)	8 (3-24)	<.001	7 (3-21)	5 (3-7)	6 (3-9)	.02	5 (3-8)
Length of in-hospital stay for VTE, mean (SD), d	18.0 (35.4)	23.0 (40.5)	NA	20.5 (38.2)	6.0 (6.1)	7.0 (6.5)	NA	6.3 (6.2)
Costs per inpatient case of VTE, mean (SD), €^d^	17 218 (36 896)	22 058 (45 403)	NA	19 684 (41 519)	3826 (NA)	3767 (NA)	NA	3809

Abbreviations: DVT, deep vein thrombosis; NA, not applicable; PE, pulmonary embolism; VTE, venous thromboembolism.

^a^ Data years are cumulative (2020-2024). Source: Research Data Centers of the Federal Statistical Office and Statistical Offices of the Federal States.^15,16,17,18,19^

^b^ Encoded as the main or secondary diagnosis.

^c^ Percentages are not reported here because the exact numbers are not allowed to be reported due to the database’s confidentiality guidelines.

^d^ To convert euros to US dollars, multiply by 1.17.

Among inpatient cases with VTE, DVT of the lower extremities was the most common location in both sexes (female, 22.4% [1546 of 6907] vs male, 16.5% [1188 of 7201]; *P* < .001), followed by intracranial DVT (female, 15.7% [1085 of 6907] vs male, 17.8% [1279 of 7201]; *P* = 001), DVT of the upper extremities (female, 15.0% [1037 of 6907] vs male, 15.8% [1137 of 7201]; *P* = .21), DVT of the visceral veins (female, 11.1% [767 of 6907] vs male, 12.3% [884 of 7201]; *P* = .03), and DVT of the inferior vena cava (female, 9.4% [649 of 6907] vs male, 9.4% [680 of 7201]; *P* = .95) (Table 1).^15,16,17,18,19^ In 21.5% of cases (3040 of 14 108), DVT was associated with catheter use, with a higher proportion among male cases (male, 23.6% [1702 of 7201] vs female, 19.4% [1338 of 6907]) (Table 1)^15,16,17,18,19^ and at a younger age (≤4 years, 27.4% [1497 of 5454]; 5-14 years, 26.6% [999 of 3756]; 15-19 years, 11.1% [544 of 4898]) (Table 2).^15,16,17,18,19^ Whereas 92.4% of VTE cases (13 038 of 14 108) were associated with DVT alone, accompanying PE was encoded as the main or secondary diagnosis for a total of 1564 inpatients (female, 13.7% [948 of 6907] and male, 8.6% [616 of 7201]; *P* < .001). The frequency of PE increased with age, from 3.0% (162 of 5454) among cases aged 4 years or younger to 5.3% (200 of 3756) among cases aged 5 to 14 years, and to 24.5% (1202 of 4898) among cases aged 15 to 19 years.

**Table 2.** Baseline Characteristics in VTE Per Age Category^a^

Characteristic	Aged ≤4 y				Aged 5-14 y				Aged 15-19 y			
	Female	Male	*P* value	Total	Female	Male	*P* value	Total	Female	Male	*P* value	Total
VTE, No. (%)^b^	2327 (42.7)	3127 (57.3)	NA	5454 (100)	1724 (45.9)	2032 (54.1)	NA	3756 (100)	2856 (58.3)	2042 (41.7)	NA	4898 (100)
VTE characteristics												
DVT lower extremity, No. (%)	225 (9.7)	284 (9.1)	.49	509 (9.3)	248 (14.4)	296 (14.6)	.91	544 (14.5)	1073 (37.6)	608 (29.8)	<.001	1681 (34.3)
DVT upper extremity, No. (%)	348 (15.0)	433 (13.8)	.26	781 (14.3)	331 (19.2)	320 (15.7)	.006	651 (17.3)	358 (12.5)	384 (18.8)	<.001	742 (15.1)
DVT inferior vena cava, No. (%)	412 (17.7)	433 (13.8)	<.001	845 (15.5)	97 (5.6)	124 (6.1)	.58	221 (5.9)	140 (4.9)	123 (6.0)	.10	263 (5.4)
DVT visceral veins, No. (%)	345 (14.8)	473 (15.1)	.79	818 (15.0)	266 (15.4)	288 (14.2)	.30	554 (14.7)	156 (5.5)	123 (6.0)	.44	279 (5.7)
DVT other veins, No. (%)	150 (6.4)	204 (6.5)	.95	354 (6.5)	81 (4.7)	104 (5.1)	.61	185 (4.9)	108 (3.8)	108 (5.3)	.01	216 (4.4)
DVT intracranial, No. (%)	377 (16.2)	628 (20.1)	<.001	1005 (18.4)	306 (17.7)	424 (20.9)	.02	730 (19.4)	402 (14.1)	227 (11.1)	<.001	629 (12.8)
DVT catheter associated, No. (%)	650 (27.9)	847 (27.1)	.51	1497 (27.4)	439 (25.5)	560 (27.6)	.16	999 (26.6)	249 (8.7)	295 (14.4)	<.001	544 (11.1)
Postthrombotic syndrome, No. (%)	0	0	NA	0	0	3 (0.1)	.31	3 (0.1)	15 (0.5)	5 (0.2)	.20	20 (0.4)
DVT alone, No. (%)	2270 (97.6)	3056 (97.7)	.73	5326 (97.7)	1661 (96.3)	1969 (96.9)	.40	3630 (96.6)	2316 (81.1)	1766 (86.5)	<.001	4082 (83.3)
PE, No. (%)^b^	71 (3.1)	91 (2.9)	.82	162 (3.0)	86 (5.0)	114 (5.6)	.44	200 (5.3)	791 (27.7)	411 (20.1)	<.001	1202 (24.5)
Patient characteristics												
Age, mean (SD), y	1.0 (1.4)	0.9 (1.3)	NA	0.9 (1.4)	9.8 (3.1)	9.7 (3.0)	NA	9.7 (3.0)	17.3 (1.4)	17.2 (1.4)	NA	17.3 (1.4)
Age, median (IQR), y	0 (0-2)	0 (0-2)	.004	0 (0-2)	10 (5-15)	10 (5-15)	.009	10 (5-15)	17 (16-19)	17 (16-19)	<.001	17 (16-19)
Cases aged <1 y, No. (%)	1361 (58.5)	1950 (62.4)	.004	3311 (60.7)	NA	NA	NA	NA	NA	NA	NA	NA
Codiagnoses												
Obesity, No. (%)	<3	<3	NA	<3	29 (1.7)	31 (1.5)	.80	60 (1.6)	140 (4.9)	93 (4.6)	.62	233 (4.8)
Cancer, No. (%)	242 (10.4)	304 (9.7)	.44	546 (10.0)	349 (20.2)	462 (22.7)	.07	811 (21.6)	300 (10.5)	385 (18.9)	<.001	685 (14.0)
Thrombophilia, No. (%)	256 (11.0)	338 (10.8)	.86	594 (10.9)	110 (6.4)	142 (7.0)	.50	252 (6.7)	262 (9.2)	197 (9.6)	.61	459 (9.4)
Kidney insufficiency, No. (%)	86 (3.7)	118 (3.8)	.94	204 (3.7)	57 (3.3)	54 (2.7)	.28	111 (3.0)	65 (2.3)	69 (3.4)	.02	134 (2.7)
Heart failure, No. (%)	261 (11.2)	303 (9.7)	.07	564 (10.3)	108 (6.3)	144 (7.1)	.35	252 (6.7)	129 (4.5)	112 (5.5)	.14	241 (4.9)
Sepsis, No. (%)	172 (7.4)	196 (6.3)	.11	368 (6.7)	80 (4.6)	97 (4.8)	.91	177 (4.7)	76 (2.7)	128 (6.3)	<.001	204 (4.2)
Polytrauma, No. (%)	<3	3	NA	4	<3	<3	NA	<3	3 (0.1)	4 (0.2)	.66	7 (0.1)
Congenital venous malformation, No. (%)	131 (5.6)	110 (3.5)	<.001	241 (4.4)	43 (2.5)	49 (2.4)	.10	92 (2.4)	25 (0.9)	41 (2.0)	.001	66 (1.3)
Chromosomal abnormalities, No. (%)	75 (3.2)	104 (3.3)	.89	179 (3.3)	66 (3.8)	45 (2.2)	.005	111 (3.0)	53 (1.9)	30 (1.5)	.36	83 (1.7)
COVID-19, No. (%)	47 (2.0)	82 (2.6)	.17	129 (2.4)	52 (3.0)	62 (3.1)	1	114 (3.0)	144 (5.0)	106 (5.2)	.87	250 (5.1)
Economic data												
Length of in-hospital stay VTE, median (IQR), d	15 (5-41)	15 (5-41)	.34	15 (5-14)	5 (1-12)	5 (1-14)	.59	5 (1-13)	5 (2-9)	6 (2-15)	<.001	6 (2-12)
Length of in-hospital stay VTE, mean (SD), d	32.6 (50.3)	32.8 (49.0)	NA	32.7 (49.6)	13.1 (24.7)	14.3 (28.6)	NA	13.8 (26.9)	9.8 (18.7)	15.3 (31.9)	NA	12.1 (25.2)
Costs per inpatient case VTE, mean, €^c^	33 481 (50 665)	34 132 (54 882)	NA	33 854 (53 120)	12 256 (27 737)	13 923 (34 862)	NA	13 156 (31 789)	6891 (19 851)	11 448 (32 069)	NA	8780 (25 728)

Abbreviations: DVT, deep vein thrombosis; NA, not applicable; PE, pulmonary embolism; VTE, venous thromboembolism.

^a^ Data years are cumulative (2020-2024). Source: Research Data Centers of the Federal Statistical Office and Statistical Offices of the Federal States.^15,16,17,18,19^

^b^ Encoded as the main or secondary diagnosis.

^c^ To convert euros to US dollars, multiply by 1.17.

Regarding patients’ risk profiles, cancer was the most common comorbidity, observed in 12.9% of female cases with VTE (891 of 6907) and 16.0% of male cases with VTE (115 of 7201) (*P* < .001) (Table 1).^15,16,17,18,19^ The frequency of cancer differed by age group, being most commonly codiagnosed in VTE cases aged 5 to 14 years (21.6% [811 of 3756] vs 10.0% [546 of 5454] among VTE cases aged ≤4 years and 14.0% [685 of 4898] among VTE cases aged 15-19 years) (Table 2).^15,16,17,18,19^ Other significant risk factors among the VTE cohort were thrombophilia (9.3% [1305 of 14 108]), chronic heart failure (7.5% [1057 of 14 108]), and sepsis (5.3% [749 of 14 108]), all with the highest incidence among the youngest age group (≤4 years). However, congenital venous malformation, chromosomal abnormalities, kidney insufficiency, obesity, and multiple trauma were relatively rare (Table 1).^15,16,17,18,19^ COVID-19 illness was diagnosed in 3.5% of VTE cases (493 of 14 108).

**Figure 1.**
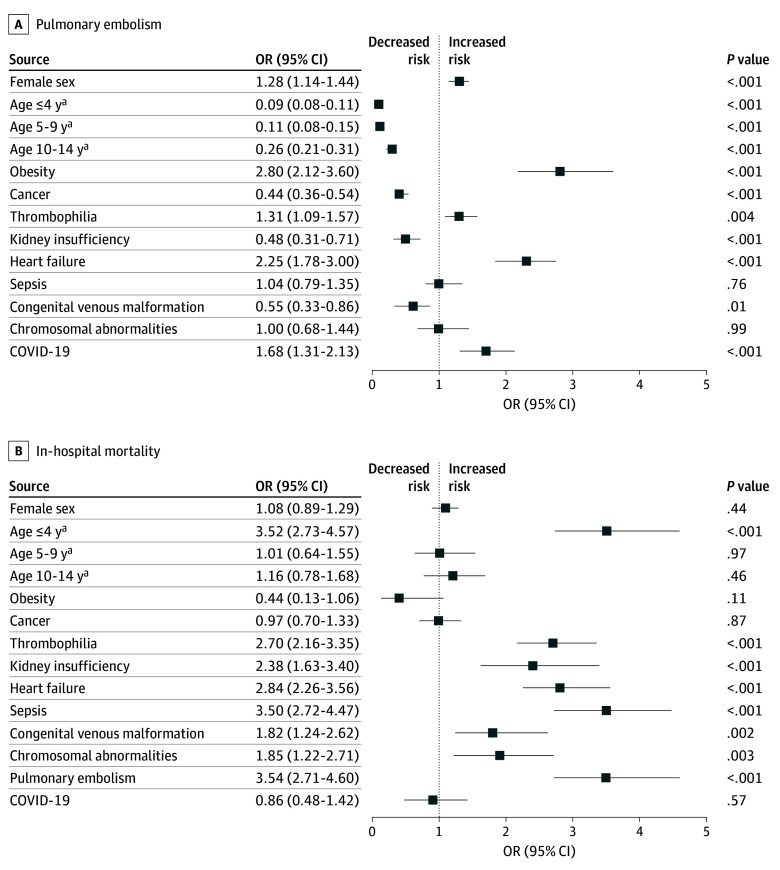
Forest Plots Showing Logistic Regression Analysis of Venous Thromboembolism (VTE) Cases Logistic regression analysis of VTE cases, illustrating the association of risk factors with the occurrence of pulmonary embolism (A) and in-hospital mortality (B) in the VTE cohort. Source: Research Data Centers of the Federal Statistical Office and Statistical Offices of the Federal States.^15,16,17,18,19^ OR indicates odds ratio. ^a^Reference = age group 15 to 19 years.

Risk factors that were independently associated with the occurrence of PE in the VTE cohort were obesity (OR, 2.80 [95% CI, 2.12-3.60]; *P* < .001), heart failure (OR, 2.25 [95% CI, 1.78-3.00]; *P* < .001), COVID-19 (OR, 1.68 [95% CI, 1.31-2.13]; *P* < .001), thrombophilia (OR, 1.31 [95% CI, 1.09-1.57]* P* = .004), and female sex (OR, 1.28 [95% CI, 1.14-1.44]; *P* < .001) (Figure 1A).^15,16,17,18,19^

**Figure 2.**
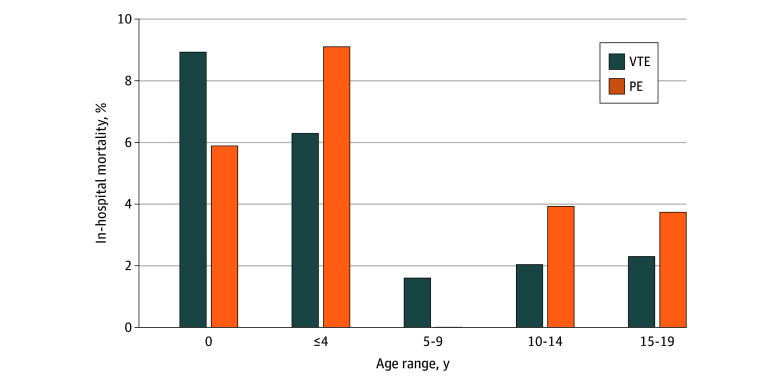
Bar Graph Showing In-Hospital Mortality of Venous Thromboembolism (VTE) and Pulmonary Embolism (PE) Inpatient Cases In-hospital mortality rates among inpatient cases with VTE as the main or secondary diagnosis and among inpatient cases with a primary diagnosis of PE. In-hospital mortality is presented by patient age groups as indicated; in addition, cases younger than 1 year are displayed separately. Data years are cumulative from 2020 to 2024. Source: Research Data Centers of the Federal Statistical Office and Statistical Offices of the Federal States.^15,16,17,18,19^

In-hospital mortality in the VTE cohort was 3.7% (521 of 14 108), decreasing with increasing age (Figure 2).^15,16,17,18,19^ In the VTE cohort, mortality risk was independently increased among children aged 4 years or younger (OR, 3.52 [95% CI, 2.73-4.57]; *P* < .001) and those with kidney failure (OR, 2.38 [95% CI, 1.63-3.40]; *P* < .001), heart failure (OR, 2.84 [95% CI, 2.26-3.56]; *P* < .001), thrombophilia (OR, 2.70 [95% CI, 2.16-3.35]; *P* < .001), sepsis (OR, 3.50 [95% CI, 2.72-4.47]; *P* < .001), and congenital venous malformation (OR, 1.82 [95% CI, 1.24-2.62]; *P* = .002) (Figure 1B).^15,16,17,18,19^ The presence of PE was associated with a 3.54-fold increased risk of in-hospital mortality among children and adolescents with VTE (OR, 3.54 [95% CI, 2.71-4.60]; *P* < .001). COVID-19 illness was not associated with increased in-hospital mortality in pediatric VTE cases.

### PE Cohort

In the subcohort with PE as the underlying cause of hospitalization (main diagnosis [n = 888]), 70.3% of cases (624 of 888) were female (Table 1).^15,16,17,18,19^ The median patient age was 18 years (IQR, 16-19 years), and only 17 of 888 cases (1.9%) were younger than 1 year (female, 1.3% [8 of 624] vs male, 3.4% [9 of 264]; *P* = .06). Thrombophilia was coprevalent in 18.6% of male cases with PE (49 of 264) compared with only 7.4% in female cases with PE (46 of 624) (*P* < .001). Other risk factors in the subcohort with PE included obesity (9.8% [87 of 888]) and chronic heart failure (8.3% [74 of 888]).

With regard to in-hospital complications, 28.4% of female cases with PE (177 of 624) vs 22.3% of male cases with PE (59 of 264) experienced acute cor pulmonale (*P* = .08) (Table 3).^15,16,17,18,19^ However, resuscitation (3.7% [33 of 888]) and cardiac arrest (5.1% [45 of 888]) remained relatively rare events. Systemic thrombolysis was performed in 7.7% of PE cases (68 of 888), and endoluminal procedures were performed in 4.5% (40 of 888). Major bleeding complication occurred in 5.0% of PE cases (44 of 888), and 19.3% (171 of 888) were receiving preexisting long-term anticoagulation therapy.

**Table 3.** Procedures and Outcomes in PE^a^

Procedure or outcome	Sex, No. (%)		*P* value	Total, No. (%)
	Female	Male		
PE main diagnosis	624 (70.3)	264 (29.7)	NA	888 (100)
Procedures				
Intensive care	29 (4.6)	16 (6.1)	.48	45 (5.1)
Invasive ventilation	8 (1.3)	15 (5.7)	<.001	23 (2.6)
Systemic thrombolysis	52 (8.3)	16 (6.1)	.30	68 (7.7)
Endoluminal procedures	27 (4.3)	13 (4.9)	.69	40 (4.5)
Anticoagulation, preexisting long-term therapy	111 (17.8)	60 (22.7)	.11	171 (19.3)
CT of thorax with contrast medium	411 (65.9)	172 (65.2)	.90	583 (65.7)
Complications				
Acute cor pulmonale	177 (28.4)	59 (22.3)	.08	236 (26.6)
Cardiac arrest	31 (5.0)	14 (5.3)	.97	45 (5.1)
Acute kidney failure	12 (1.9)	7 (2.7)	.67	19 (2.1)
Major bleeding complication	32 (5.1)	12 (4.5)	.84	44 (5.0)
Resuscitation	23 (3.7)	10 (3.8)	>.99	33 (3.7)
In-hospital mortality	24 (3.8)	10 (3.8)	>.99	34 (3.8)

Abbreviations: CT, computed tomography; NA, not applicable; PE, pulmonary embolism.

^a^ Data years are cumulative (2020-2024). Source: Research Data Centers of the Federal Statistical Office and Statistical Offices of the Federal States.^15,16,17,18,19^

In-hospital mortality was observed in 3.8% of PE cases (34 of 888) with no significant difference between the sexes (Table 3).^15,16,17,18,19^ The highest PE mortality rate was seen among children aged 4 years or younger (9.1% [due to the small number of cases, the number is not permitted to be reported in accordance with DESTATIS confidentiality guidelines]) compared with older children and adolescents (Figure 2).^15,16,17,18,19^

In terms of economic burden, the mean (SD) length of hospital stay was 20.5 (38.2) days in the VTE cohort and 6.3 (6.2) days for cases with a primary diagnosis of PE (Table 1).^15,16,17,18,19^ The mean (SD) cost was €19 684 (€41 519) per inpatient VTE case, and health expenses were particularly high for the youngest patients, aged 4 years or younger (€33 854 [€53 120] per inpatient case) (to convert euros to US dollars, multiply by 1.17.) (Table 2).^15,16,17,18,19^ In the PE cohort, the mean (SD) cost was €3826 (SD not available) for female patients and €3767 (SD not available) for male patients (Table 1 and Table 2).^15,16,17,18,19^

## Discussion

This German cohort study identified 14 108 pediatric inpatient cases of VTE over a 5-year period, corresponding to a hospital-based incidence of 15.3 per 10 000 pediatric inpatient cases per year. On an international level, compared with hospital-based incidences of 5.3 per 10 000 pediatric hospital admissions in Canada and up to 106 VTE cases per 10 000 pediatric admissions in the US,^7,20,21,22^ the in-hospital incidence of VTE among children and adolescents in Germany is toward the lower end of that range. Compared with population-based incidences of VTE in children and adolescents of approximately 1.4 to 5.1 per 100 000 children,^3,4,5,6^ the hospitalization rate is approximately 30- to 100-fold increased, suggesting a marked demand for inpatient care.

On the one hand, VTE itself may frequently require hospitalization, particularly for younger children. VTE cases in early childhood represent a major share of the inpatient cohort in our study (VTE at age <1 year, 23.5%; compared with 32% in administrative data from the US^22^ and 18% in registry data from Canada^7^). On the other hand, hospitalized pediatric patients are more likely to have conditions and undergo treatments that predispose one to develop VTE. Among very young patients, VTE is observed to be mostly accompanied by severe clinical conditions requiring substantial health care resources (eg, intensive care unit treatment, long hospital length of stay, and high costs). Organ failure, sepsis, or congenital medical conditions define complex clinical courses among young patients with VTE more than in older age groups, which supports previous observations derived from small studies.^12^ Although thrombophilia was seen at equal rates among men and women with VTE, our study found that thrombophilia was present in 18.6% of male patients with PE but only 7.4% of female patients with PE. Although thrombophilia is a congenital risk factor and its prevalence in VTE cases remains relatively constant across all age groups, its association with the development of PE is primarily seen among young adults. Moreover, thrombophilia was independently associated with a 2.7-fold increased risk of in-hospital mortality. Furthermore, although polytrauma is commonly reported as a VTE risk factor,^7^ it was not detected at relevant frequencies in our VTE cohort by sex or age group. In contrast, cancer, one of the main comorbidities in VTE, was most prevalent among children aged 5 to 14 years, affecting 21.6% of cases. In line with this finding, the proportion of catheter-associated DVT is almost one-third of VTE cases among patients aged 14 years or younger, yet it could be avoided. Most likely, because catheter-associated DVT is considered to pose a relatively low risk for PE,^23^ cancer was associated with a decreased PE risk in the logistic regression analysis.

Conversely, among patients older than 14 years, a high proportion of DVT of the lower extremities was observed, accompanied by risk profiles that increasingly resembled that of adult patients.^6,8^ Here, increased risk of VTE recurrence^24^ may further accumulate to high hospitalization numbers in late adolescence among our cohort. The disproportionate increase in VTE cases among female patients may indicate an increasing association of hormonal and lifestyle-modifying factors with VTE development among girls and young women from puberty onward. In addition, we also found a substantial risk of PE among cases aged 15 to 19 years compared with younger age groups. Given the importance of COVID-19 during the survey period, COVID-19 illness was found to be associated with the development of PE (OR, 1.68), but not with in-hospital mortality.

Overall, PE was observed in 11.1% of VTE cases, comparable to pediatric inpatient data from the US (9.1%; data years 2008-2019^22^). The significant preponderance of inpatient cases of PE among female children and adolescents is surprising in view of a commonly very balanced sex ratio in nonselected adult PE cohorts.^25,26^ A possible explanation could be the association of sex with the presence and weighting of PE-specific risk factors differently, as reported for adults.^27^ Given the results of the adjusted analysis, heart failure was particularly shown to be associated with an increased risk of PE, and the presence of acute cor pulmonale was increased among female patients compared with male patients. However, the in-hospital mortality rate remained less than 4% for either sex. In an international comparison, the German in-hospital mortality rates for VTE and PE were slightly higher compared with Canada (30-day case fatality rates, 2.0% for VTE and 1.9% for PE among patients aged ≤19 years^1^) but relatively lower compared with the US (8% for VTE among patients aged ≤18 years^21^). The risk of in-hospital mortality among children aged 4 years or younger was increased 3.5-fold compared with adolescents aged 15 to 19 years. In accordance with German registry data (case fatality rate, 9% among neonates with symptomatic VTE^6^), this finding reinforces the identification of young children with VTE as highly vulnerable and at risk for fatal clinical courses. Further research is needed to improve the prognosis and quality of health among children and adolescents with VTE.

### Strengths and Limitations

Our study has some strengths. This study used German nationwide data and represents the largest European VTE cohort of children and adolescents to date. It provides insight into disease manifestation with regard to sex at different stages of childhood development. A particular strength is the high level completeness of data due to statutory requirements (notably, the high level of accuracy of case fatality rates) as such events are required by law to be reported.

This study also has some limitations. First, the study included only cases receiving inpatient care. Although VTE can be treated on an outpatient basis, it can be assumed that very young children with VTE are likely to be admitted to the hospital; this assumption may lead to an underestimation of the medical care needs of adolescents receiving ambulatory treatment. Although VTE may not have been the main reason for hospital admission, it required treatment in all cases (irrespective of being coded as the main or secondary diagnosis) because otherwise it must not be encoded. Second, in the DESTATIS database, inpatient readmission cannot be identified, which introduces some bias to the characteristics of the cohort, particularly when compared with patient-centered analyses. Therefore, the focus of the study was to give a realistic picture of the VTE burden in the inpatient sector as well as its related health care factors. Third, case identification relied on *ICD-10-GM* codes in the DESTATIS inpatient database, which may involve miscoding. Although formal validation for VTE and PE coding is limited, administrative data generally show high specificity. Strict German reimbursement rules and mandatory coding, together with external audits by the Medical Service of the Health Insurance (reviewing approximately 10%-20% of cases), make “rule-out” diagnoses unlikely and support the reliability of recorded VTE events. A major limitation is that comprehensive information on pharmacotherapy is not included in the database. Fourth, logistic regression analysis of in-hospital mortality in the PE subcohort was not feasible due to overfitting in the model because of the relatively small number of events. Therefore, we were unable to assess the association of individual risk factors with in-hospital mortality (analogous to the VTE cohort).

## Conclusions

In this nationwide inpatient cohort study of children and adolescents, VTE was associated with notable hospitalization rates with corresponding care needs. These care needs were reflected in long hospital lengths of stay and high costs per case, particularly for very young children. For each of these 14 108 inpatient stays, a decision regarding the therapeutic regimen had to be made. Given the sparse data available, there is a need for appropriate studies to provide evidence-based support for the safety and effectiveness of medical interventions for children and adolescents with VTE.

## Notes

Supplement 1. **eFigure 1. **Flow Chart on Case Selection in the DESTATIS Data Base, Data Years 2020-2024 **eFigure 2. **VTE Inpatient Cases in Children and Adolescents—Time Trend **eFigure 3. **Panel A: VTE Inpatient Cases per Sex and Age. The Image Shows All German Nationwide Inpatient Cases With Venous Thromboembolic Event (VTE) as Main or Secondary Diagnosis in Children and Adolescent Patients Aged 0-19 Years, Separated for Patient Age in Male (Blue Bars) Versus Female (Orange Bars) Cases. Panel B: Subgroup of Inpatient Cases With Pulmonary Embolism (PE; as Main or Secondary Diagnosis) Per Sex and Age Groups Accordingly **eTable. **ICD-10 GM and OPS Codes

Supplement 2.
Data Sharing Statement
