# Supplementary material for: Inpatient Characteristics and Outcomes of Venous Thromboembolism Among Children and Adolescents
Source: JAMA Netw Open. 2026 Jun 9;9(6):e2617459. doi: 10.1001/jamanetworkopen.2026.17459 (PMC13250709; doi:10.1001/jamanetworkopen.2026.17459)
Supplement: Supplement 1. — eFigure 1. Flow Chart on Case Selection in the DESTATIS Data Base, Data Years 2020-2024 eFigure 2. VTE Inpatient Cases in Children and Adolescents—Time Trend eFigure 3. Panel A: VTE Inpatient Cases per Sex and Age. The Image Shows All German Nationwide Inpatient Cases With Venous Thromboembolic Event (VTE) as Main or Secondary Diagnosis in Children and Adolescent Patients Aged 0-19 Years, Separated for Patient Age in Male (Blue Bars) Versus Female (Orange Bars) Cases. Panel B: Subgroup of Inpatient Cases With Pulmonary Embolism (PE; as Main or Secondary Diagnosis) Per Sex and Age Groups Accordingly eTable. ICD-10 GM and OPS Codes [file jamanetwopen-e2617459-s001.pdf]

Supplementary Online Content

Friebe S, Scheinert D, Tokur Sonuvar E, Kirsten T, Freisinger E. Inpatient characteristics and outcomes of venous thromboembolism among children and adolescents. *JAMA Netw Open.* 2026;9(6):e2617459. doi:10.1001/jamanetworkopen.2026.17459

eFigure 1. Flow Chart on Case Selection in the DESTATIS Data Base, Data Years 2020-2024
eFigure 2. VTE Inpatient Cases in Children and Adolescents—Time Trend
eFigure 3. Panel A: VTE Inpatient Cases per Sex and Age. The Image Shows All German Nationwide Inpatient Cases With Venous Thromboembolic Event (VTE) as Main or Secondary Diagnosis in Children and Adolescent Patients Aged 0-19 Years, Separated for Patient Age in Male (Blue Bars) Versus Female (Orange Bars) Cases. Panel B: Subgroup of Inpatient Cases With Pulmonary Embolism (PE; as Main or Secondary Diagnosis) Per Sex and Age Groups Accordingly
eTable. ICD-10 GM and OPS Codes

This supplementary material has been provided by the authors to give readers additional information about their work.

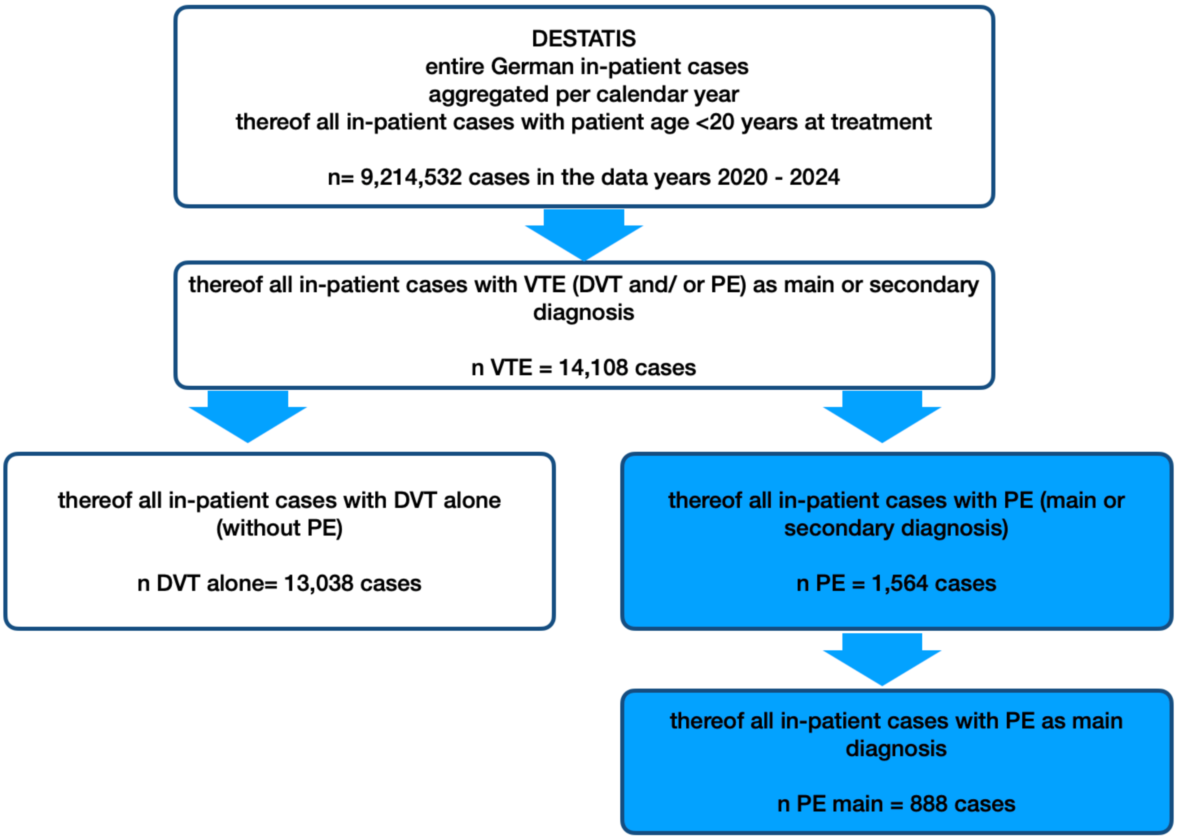

eFigure 1. Flow Chart on Case Selection in the DESTATIS Data Base, Data Years 2020-2024

© 2026 Friebe S et al. *JAMA Network Open.*

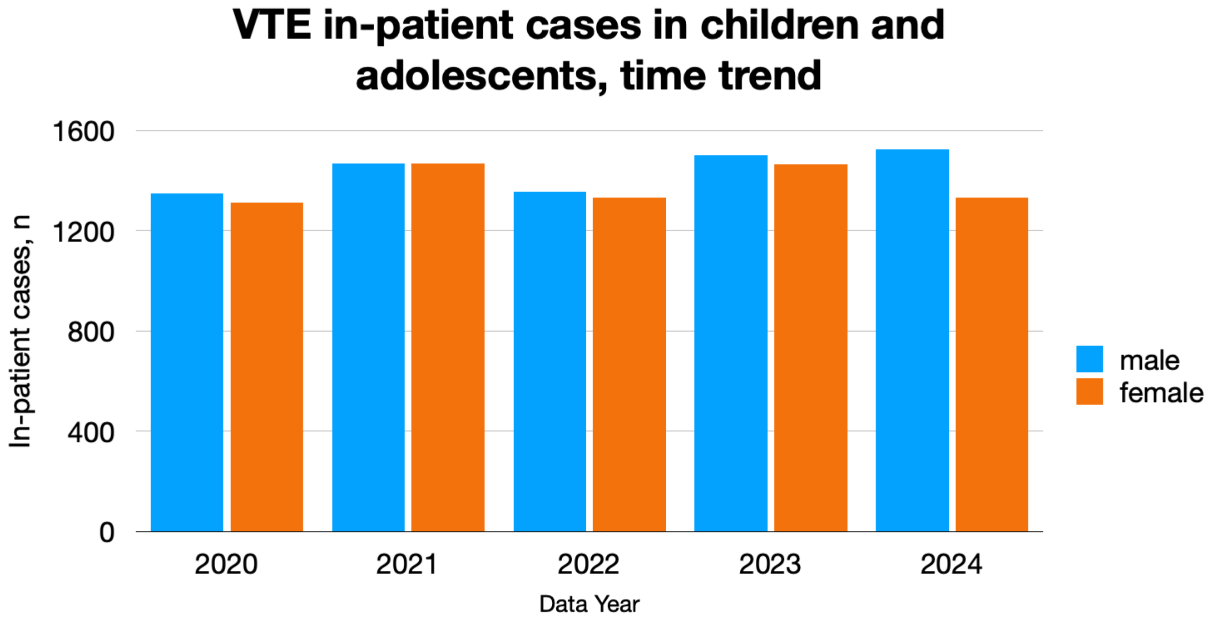

eFigure 2. VTE Inpatient Cases in Children and Adolescents—Time Trend. The image shows all German nationwide in-patient cases with VTE as main or secondary diagnosis in children and adolescent patients aged 0-19 years, separated for male (blue bars) versus female (orange bars) sex over a five-year time period (2020 - 2024).

Source: RDC of the Federal Statistical Office and Statistical Offices of the Federal States, DOI: [10.21242/23141.2020.00.00.6.1.0, 10.21242/23141.2021.00.00.6.1.0, 10.21242/23141.2022.00.00.6.1.0, 10.21242/23141.2023.00.00.6.1.0, 10.21242/23141.2024.00.00.6.1.0], own calculations.

© 2026 Friebe S et al. *JAMA Network Open.*

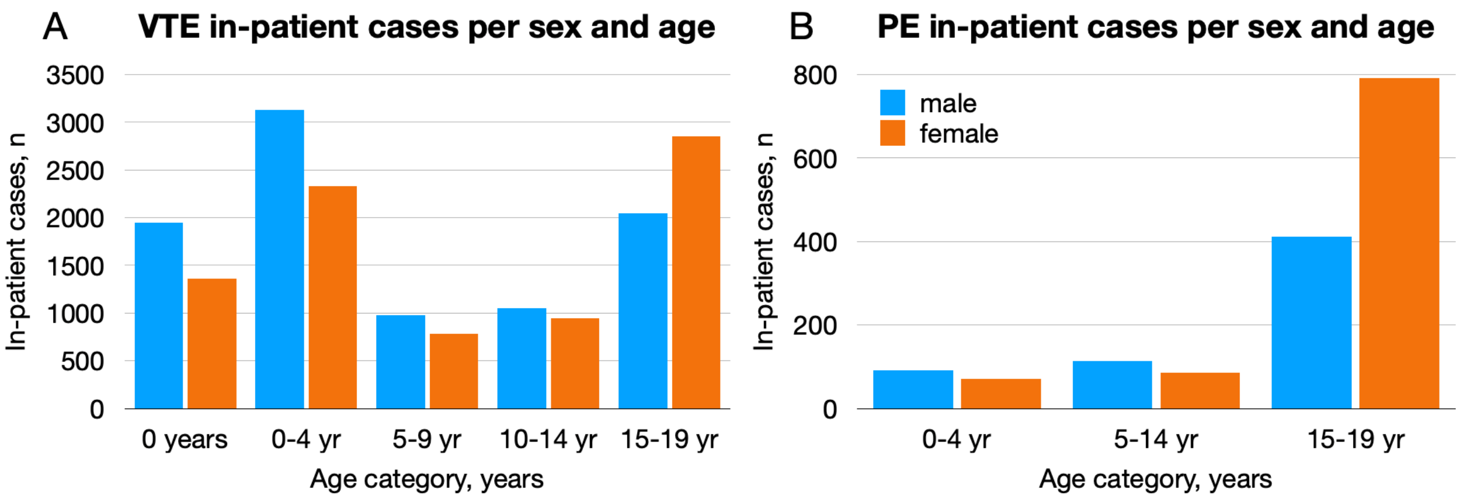

eFigure 3. Panel A: VTE Inpatient Cases per Sex and Age. The Image Shows All German Nationwide Inpatient Cases With Venous Thromboembolic Event (VTE) as Main or Secondary Diagnosis in Children and Adolescent Patients Aged 0-19 Years, Separated for Patient Age in Male (Blue Bars) Versus Female (Orange Bars) Cases. Panel B: Subgroup of Inpatient Cases With Pulmonary Embolism (PE; as Main or Secondary Diagnosis) Per Sex and Age Groups Accordingly. Data years cumulative 2020 - 2024.

Source: RDC of the Federal Statistical Office and Statistical Offices of the Federal States, DOI: [10.21242/23141.2020.00.00.6.1.0, 10.21242/23141.2021.00.00.6.1.0, 10.21242/23141.2022.00.00.6.1.0, 10.21242/23141.2023.00.00.6.1.0, 10.21242/23141.2024.00.00.6.1.0], own calculations.

© 2026 Friebe S et al. *JAMA Network Open.*

eTable. ICD-10 GM and OPS Codes

Diagnosis	ICD-10 GM Codes
**VTE characteristics**	
DVT, all	I80.1, I80.20, I80.28, I80.3, I80.81, I82.81, I82.2, I81, I82.0, I82.3, I82.80, I82.88, I82.9, T82.8, G08, I67.6, I63.6
DVT lower extremity	I80.1, I80.20, I80.28, I80.3
DVT upper extremity	I80.81, I82.81
DVT inferior vena cava	I82.2
DVT visceral veins	I81, I82.0, I82.3, I82.80
DVT intracranial	G08, I67.6, I63.6
DVT other veins	I82.88, I82.9
DVT catheter associated	T82.8
PE	I26.0, I26.9
**Co-Diagnoses**	
Obesity	E65, E66.-, E68
Cancer	C00-C97
Thrombophilia	D65.-, D68.5, D68.6, E72.1
Renal insufficiency	N18.-, P96.0, N04,-
Heart failure	I50.-
Sepsis	A41, R57.2
(Poly-)trauma	T00-T07
Congenital venous malformation	Q26.-
Chromosomal abnormalities	Q90-Q99
Acute cor pulmonale	I26.0
Cardiac arrest	U69.13!, I46.0, I46.1, I46.9
Acute kidney disease	N17
Major bleeding complication	**ICD:** I60, I61, I62, S06.33, S06.34, S06.4, S06.5, S06.6, G95.1, H43.1, H45.0 , H35.6, K66.1, I31.2, M25.0, M62.2<br>**OPS:** 8-800.0,8-800.1, 8-800.c
Post-thrombotic syndrome	I87.0
COVID-19	U07.1!, U07.2!, U09.9!, U08.9, U10.9
**Procedures**	
Anticoagulation, pre-existing long-term therapy	Z.92.1
	**OPS Codes**
Resuscitation	8-77
Intensive care	8-98d
Invasive ventilation	8-711, 8-712
Systemic thrombolysis	8-020.8
Endoluminal procedures	8-838.00, 8-838.10, 8-838.20, 8-838.30, 8-838.40, 8-838.50, 8-838.60, 8-838.70, 8-838.d0, 8-838.c0, 8-838.n0, 8-838.x0, 8-839.10, 8-839.11, 8-839.3
CT thorax with contrast medium	3-222

© 2026 Friebe S et al. *JAMA Network Open.*

eTable. ICD-10 GM and OPS Codes. The table lists all codes for diagnoses (ICD-10 GM) and procedures (OPS) that have been used in the analysis.

© 2026 Friebe S et al. *JAMA Network Open.*
