# Supplementary material for: Inpatient Characteristics and Outcomes of Venous Thromboembolism Among Children and Adolescents
Source: JAMA Netw Open. 2026 Jun 9;9(6):e2617459. doi: 10.1001/jamanetworkopen.2026.17459 (PMC13250709; doi:10.1001/jamanetworkopen.2026.17459)
Supplement: Supplement 2. — Data Sharing Statement [file jamanetwopen-e2617459-s002.pdf]

Data Sharing Statement

Friebe. Inpatient Characteristics and Outcomes of Venous Thromboembolism Among Children and Adolescents. *JAMA Netw Open*. Published June 09, 2026.
doi:10.1001/jamanetworkopen.2026.17459

Data
Data available: Yes
Data types: Other (please specify)
Additional Information: Coding list (ICD-10 GM, OPS) The deidentified original data can be accessed via the DESTATIS - charges apply.
How to access data: The coding list will be made available as online supplementary table.
When available: With publication

Supporting Documents
Document types: Statistical/analytic code
How to access documents: The coding list will be made available as online supplementary table.
When available: With publication

Additional Information
Who can access the data: to all readers of the published manuscript
Types of analyses: for any purpose
Mechanisms of data availability: free release as supplementary data
Any additional restrictions: none.
